# Population-based study suggests an increased risk of Alzheimer’sdisease in Sjögren’s syndrome

**DOI:** 10.1007/s10067-017-3940-y

**Published:** 2017-12-14

**Authors:** Po-Chou Liliang, Cheng-Loong Liang, Kang Lu, San-Nan Yang, Meng-Tsang Hsieh, Yi-Cheng Tai, Kuo-Wei Wang

**Affiliations:** 1 Department of Neurosurgery, E-Da Cancer Hospital, I-Shou University, No.1, Yida Road, Jiaosu Village, Yanchao District, Kaohsiung, 82445 Taiwan; 2 Department of Neurosurgery, E-Da Hospital, I-Shou University, Kaohsiung, Taiwan; 3 School of Medicine for International Students, I-Shou University, Kaohsiung, Taiwan; 4 Department of Neurology, E-Da Hospital, I-Shou University, Kaohsiung, Taiwan

**Keywords:** Alzheimer’s disease, Epidemiology, Sjögren’s syndrome

## Abstract

This population-based study was designed to estimate and compare the risk of Alzheimer’s disease (AD) between patients with primary Sjögren’s syndrome (SS) and non-SS patients during a 10-year follow-up period. This is a retrospective cohort study. Data were obtained from the Taiwan’s National Health Insurance Research Database. We identified 4463 primary SS patients and 22,315 non-SS patients; patients were matched by sex, age, and the year of index use of health care. Each patient was studied to identify the subsequent manifestation of AD. Cox proportional hazard regression was used to study the subsequent manifestation of AD, and Kaplan-Meier survival curves were used to compare survival probability. During the 10-year follow-up period, 7 primary SS and 13 non-SS patients developed AD. During the 10-year follow-up period, the risk of AD was 2.68-fold higher in the primary SS cohort with an overall adjusted hazard ratio (HR) of 2.69 (95% CI 1.07–6.76), after adjusting for demographics and comorbidities. Within the 10-year period, patients with primary SS showed a 2.69-fold increased risk of developing AD. This risk increases with time, and the relative risk of AD is higher in older patients with primary SS.

## Introduction

Sjögren’s syndrome (SS) is a relatively common systemic autoimmune rheumatic disease, in which lymphocytic infiltration of salivary and lacrimal glands leads to immune-mediated secretory dysfunction. The resulting dryness of the mouth and eyes is termed “sicca syndrome.” SS is referred to as “primary” in patients who do not have an additional systemic rheumatic disease, and “secondary” when immune-mediated sicca syndrome coexists in patients with systemic lupus erythematosus (SLE), scleroderma, rheumatoid arthritis (RA), or other autoimmune rheumatic diseases [1]. Systemic involvement (extraglandular manifestations) is common and often results in potentially life-threatening complications, such as lymphoma [2, 3]. Neuropsychiatric complications, including headache, cognitive dysfunction, and mood disorders, are reported in approximately 20% of SS patients [4, 5].

Recently, an association was noted between neuropsychiatric symptoms and autoimmune diseases [4]. Previous studies indicate that inflammatory mechanisms may play an important role in increasing the risk of cognitive impairment and stroke [4–7]. Most of those studies focused on rheumatoid arthritis (RA) or systemic lupus erythematosus (SLE) or described an increased risk of cardiovascular disease and autoimmune dementia [6–13].

This nationwide, population-based study was designed to estimate and compare the risk of Alzheimer’s disease (AD) between primary SS patients and non-SS patients during a 10-year period that followed primary SS diagnosis. We were interested in determining whether medical comorbidities or primary SS might explain such an association.

## Methods

### Database/standard protocol approvals, registrations, and patient consents

This study was a retrospective cohort study. The study cohort included one primary SS patient group and control group without SS but with similar comorbidities. The patients and controls used in our study were selected from Taiwan’s National Health Insurance Research Database (NHIRD) [14]. NHIRD was obtained from the National Health Research Institutes (NHRI), which is maintained by the Bureau of National Health Insurance, Taiwan. NHIRD contains non-identifying secondary data that scientists can access for research purposes. Details on database generation, monitoring, and maintenance are published online by the Taiwan National Health Research Institutes (http://nhird.nhri.org.tw/). This study was exempted from full review by the institutional review board.

### Study sample

SS patients were defined as those with catastrophic illness registration cards and who met the criteria defined by the International Classification of Diseases, Ninth Revision, Clinical Modification (ICD-9-CM), code (710.2 for Sjögren’s syndrome). These diagnoses had to occur between 2000 and 2005 [6]. In Taiwan, SS patients can apply for catastrophic illness registration cards from the Bureau of National Health Insurance. Individuals can qualify for catastrophic illness registration cards for SS if their clinical manifestations and laboratory data meet the criteria established by specialists in rheumatology according to the criteria proposed by the American-European Consensus Group [15] and were reviewed by rheumatologists commissioned by the NHI. Individuals with these certificates do not need to make co-payments when seeking health care for SS. Thus, the catastrophic illness patient data are highly accurate and reliable. We excluded secondary SS patients from these SS patients in this study.

In order to observe the risk of developing AD in primary SS patients, subjects who were diagnosed with AD (ICD-9-CM codes 331.0) prior to SS diagnosis were excluded from the study. A total of 4463 patients that were newly diagnosed with primary SS between 2000 and 2005 were selected for the study.

A comparison cohort was randomly selected from the patient records remaining in the NHIRD. We randomly selected five comparison subjects for each identified primary SS case. We randomly selected 22,315 individuals that were matched in terms of sex, age, similar comorbidities, and the index date for the year health care services provided. The selected comorbidities included diabetes (ICD-9 code: 250.00–250.93), hyperlipidemia (ICD-9 code: 270), hypertension (ICD-9 code: 401–405), coronary artery disease (CAD) (ICD-9-CM codes: 410–414 or 429.2), heart failure (HF) (ICD-9-CM code: 428), atrial fibrillation (AF) (ICD-9-CM code: 427.31), and stroke (ICD-9-CM codes: 430–438)].

### Statistical analysis

The primary goal of this study was to determine whether a primary SS patient had received ambulatory care visits or undergone hospitalizations for AD. We used Pearson’s *χ* ^2^ tests to compare differences between patients with and without SS in terms of demographic characteristics (i.e., residence location—northern, central, eastern, or southern Taiwan) and selected comorbidities [diabetes (ICD-9 code: 250.00–250.93), hyperlipidemia (ICD-9 code: 270), hypertension (ICD-9 code: 401–405), coronary artery disease (CAD) (ICD-9-CM codes: 410–414 or 429.2), heart failure (HF) (ICD-9-CM code: 428), atrial fibrillation (AF) (ICD-9-CM code: 427.31), and stroke (ICD-9-CM codes: 430–438)] at baseline [16, 17]. The baseline comorbidities were identified for subjects in both cohorts in the inpatient setting 1 year preceding the index dates.

Both the unadjusted and adjusted HRs for the analyses were obtained by evaluating the association between primary SS and AD during the 10-year follow-up period, after adjusting for demographic characteristics and selected comorbidities. The 10-year AD-free survival rates were estimated using the Kaplan–Meier method, which uses the log-rank test to examine differences between cohorts in dementia-free survival rates. Stratified Cox proportional hazard regressions (stratified by sex, age group, and year of index health care use) were performed to compare 10-year AD-free survival rates between the two cohorts, after adjusting for geographic region and the selected comorbidities. We also explored the relationship between primary SS and AD in different age groups. HR values along with 95% CIs were computed with a significance level of 0.05. All statistical analyses were conducted using the SAS statistical package (SAS System for Windows, V.9.2, SAS Institute Inc.) with the assistance of the Biostatistics Consulting Centre at the National Cheng Kung University Hospital.

## Results

Our study identified 4463 patients in the primary SS cohort and 22,315 patients in the non-SS cohort. The analysis of the demographic data is presented in Table 1. Men accounted for 13.5% of each cohort. After matching for gender, age, and the index year, we found no significant differences in age or sex between the primary SS and non-SS patients. The primary SS cohort, however, had a higher prevalence of hyperlipidemia (*p* < 0.0001), hypertension (*p* < 0.0001), and coronary artery disease (*p* < 0.0001). There were no significant differences in the distribution of comorbidities between the groups DM (*p* = 0.26), HF (*p* = 0.26), AF (*p* = 0.43), and stroke (*p* = 0.43).

**Table 1:** Comparison of demographic characteristics and comorbidities of primary SS and non-SS patients

	Patients with primary SS (*N* = 4463)		Comparison patients (*N* = 22,315)		
Characteristic	*N*	%	*N*	%	*p*
Sex					1.0000
Male	601	13.5	3005	13.5	
Female	3862	86.5	19,310	86.5	
Age, year					1.0000
< 36	403	9.03	2015	9.03	
36–50	1402	31.4	7010	31.4	
51–65	1599	35.8	7995	35.8	
> 65	1059	23.7	5295	23.7	
Geographic region					< 0.0001
Northern	1684	37.7	10,078	45.2	
Central	1430	32.0	4394	19.7	
Southern	1083	24.3	6699	30.0	
Eastern	266	5.96	1142	5.12	
Comorbidities					
Diabetes	174	3.90	953	4.27	0.26
Hyperlipidemia	137	3.07	424	1.90	< 0.0001
Hypertension	441	9.88	1724	7.73	< 0.0001
CAD	249	5.58	797	3.57	< 0.0001
HF	52	1.17	219	0.98	0.26
AF	30	0.67	128	0.57	0.43
Stroke	146	3.27	687	3.08	0.50

*CAD* coronary artery disease, *HF* heart failure, *AF* atrial fibrillation

**Fig. 1:**
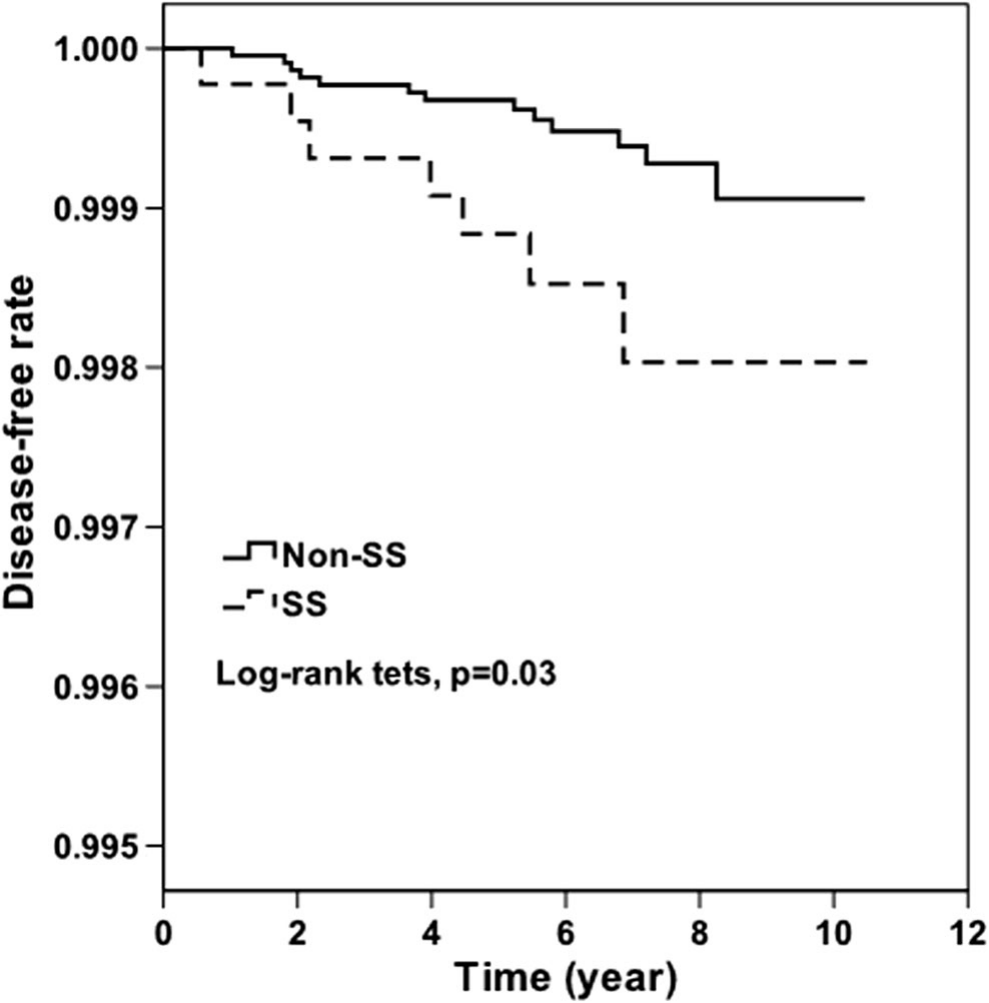
AD-free survival rates of patients with primary SS and those in the comparison cohort in Taiwan, 2000 to 2010

Table 2 shows the crude and adjusted HRs for AD during the 10-year follow-up period, after the year index between patients and controls. We found that, of the 26,778 sampled subjects, 20 patients experienced AD during the 10-year follow-up period, including 7 patients (0.16%) from the study cohort and 13 patients (0.06%) from the comparison cohort. The incidence of AD was 2.68-fold (95% CI, 1.07–6.73; *p* < 0.05) greater in the primary SS cohort than in the non-SS cohort, with an adjusted HR of 2.69 (95% CI, 1.07–6.76; *p* < 0.05). Figure 1 shows the disease-free survival curves using the Kaplan–Meier method.

**Table 2:** AD risk among sampled patients during the 10-year follow-up period from index health care utilization

	Total *N* = 26,778		Patients with primary SS *N* = 4463		Comparison patients *N* = 22,315	
AD occurrence	*N*	%	*N*	%	*N*	%
Yes	20	0.08	7	0.16	13	0.06
No	26,758	99.9	4456	99.8	22,302	99.9
Crude HR (95% CI)			2.68 (1.07–6.73)*		1.00	
Adjusted HR (95% CI)			2.69 (1.07–6.76)*		1.00	

Hazard ratio was calculated by using the Cox proportional regression method during the 10-year follow-up period. Adjustments were made for demographic characteristics (age, sex, and the geographical region) and selected comorbidities in patients (diabetes, hyperlipidemia, hypertension, coronary artery disease, heart failure, atrial fibrillation, and stroke)

*p < 0.05

Table 3 presents the prevalence HR and adjusted HR for AD between cohorts. The HR for primary SS patients older than 70 years was 3.69 (95% CI 1.28–10.6; *p* < 0.05), and an adjusted HR was 3.69 (95% CI 1.27–10.7; *p* < 0.05).

**Table 3:** Crude and adjusted hazard ratios of AD by different age groups among sampled patients during the 10-year follow-up from index health care utilization

	Total *N* = 26,778		Patients with primary SS *N* = 4463		Comparison patients *N* = 22,315	
Age group	Event no.	%	Event no.	%	Event no.	%
< 69	6	0.03	1	0.03	5	0.03
Crude HR (95% CI)			1.00 (0.12–8.56)		1.00	
Adjusted HR (95% CI)			0.95 (0.11–8.21)		1.00	
≥ 70	14	0.34	6	0.88	8	0.23
Crude HR (95% CI)			3.69 (1.28–10.6)*		1.00	
Adjusted HR (95% CI)			3.69 (1.27–10.7)*		1.00	

Hazard ratio was calculated by using stratified Cox proportional regression (stratified on sex, age group, and the year of index health care use) during the 10-year follow-up period. Adjustments were made for patients’ select comorbidities (diabetes, hyperlipidemia, hypertension, coronary artery disease, heart failure, atrial fibrillation, and stroke)

*p < 0.05

## Discussion

There have long been concerns regarding the relationship between SS and neuropsychiatric syndromes, including headache, cognitive dysfunction, mood disorder, and polyneuropathy [4]. Nevertheless, previous studies that have examined this relationship have some shortcomings, which include a small sample size, or a definitive diagnosis of AD [12, 13]. The prevalence of AD in primary SS has been unknown until now [18, 19]. To our knowledge, this is the first study that investigates the risk of AD among primary SS patients in Asia. Our retrospective, population-based cohort study, which was conducted with data obtained from Taiwan’s NHIRD, demonstrated that a primary SS diagnosis was independently associated with a 2.69-fold higher risk of subsequent AD, even after adjusting for region of residence and selected comorbidities. Compared to the non-SS cohort, crude and adjusted HRs for AD were highest in older patients (≧ 70 years old).

While cognitive symptoms are commonly experienced, very little is understood concerning the mechanisms of cognitive dysfunction in primary SS. There are several reasons why primary SS patients may have an increased risk of developing AD. Primary SS is a relatively common systemic autoimmune rheumatic disease, in which lymphocytic infiltration of salivary and lacrimal glands leads to immune-mediated secretory dysfunction [1]. In the past, diagnosis has depended on the presence of typical clinical features and/or parotid gland swelling together with focal lymphocytic infiltration demonstrated on biopsy of minor salivary glands and lips. However, serologic findings recently have been recognized to have diagnostic values. Serologic findings include autoantibodies against SS-A/Ro and SS-B/La, antinuclear antibodies, anti-salivary gland antibodies, and rheumatoid factor [20, 21]. The presence of anti-Ro (SS-A) antibodies defines a subset of patients with Sjögren’s syndrome who have systemic clinical manifestations including vasculitis, hematologic abnormalities, and serologic hyperreactivity [22]. Recently, another antibody was found and a pathophysiological role for circulating anti-muscarinic acetylcholine receptor (mAChR) autoantibodies in patients with primary SS was proposed. These autoantibodies recognized and activated mAChRs in both salivary and lacrimal glands [23]. There is a strong regulatory action of parasympathetic stimulation on the secretion of lacrimal and salivary glands [23]. Lacrimal and salivary gland mAChRs are coupled to various signaling pathways, and the production of nitric oxide is of particular interest, because it is involved in several pathologic processes, including SS, where increased levels of nitrites are found in the saliva [23] mAChRs that, acting as an “agonist-like agent,” resulted in a primary, organ-specific dysfunction. Possibly, in primary SS and secondary SS, direct mAChR antibody-mediated tissue damage might occur through nitric oxide generation and accumulation, with an adverse effect on the lacrimal glands. Immunologic generation of nitric oxide could have cytotoxic effects on the cell, through the production of free radicals [23]. This could have a special pathologic role, particularly in SS, where the increased release of inflammatory mediators could induce uncommonly high levels of nitric oxide [23]. It is known that anti-Ro/SSA and anti-La/SSB antibodies are directed against ribonucleoprotein complexes. Their localization is mainly cytoplasmic, and the increased expression of Ro52 in SS and SLE patients is probably implicated positively or negatively in the alteration of cellular immune responses and in the increased apoptosis observed in primary SS patients. Ro52 in primary SS can act as a negative regulator of interferon production, ubiquitination, and degradation of the transcription factors IRF-3, IRF-7, and IRF-8 inducing major alterations in cytokine expression and production [24]. The inflammatory response is associated with an increased risk of cognitive decline [25]. Cevimeline (M1 muscarinic agonist, AF102B) is a drug used in patients with SS to improve sicca symptoms, and it is recognized in innovative therapies for Alzheimer’s disease [26]. The M1 mAChR appears to be linked with two other major hallmarks of this disease β-amyloid (Aβ) and hyperphosphorylated τ proteins. Activation of M1 mAChR could alter beneficially Aβ and τ proteins and apolipoprotein E (ApoE), as well as some processes involving certain G-proteins and neurotrophins [26, 27]. Muscarinic agonists induce differentiation in PC12 cells stably transfected with M1 mAChR (PC12M_1_ cells), and these neurotrophic effects are synergistic with a variety of neurotrophins [26, 28]. Several studies showed that several neurotrophins (nerve growth factor [NGF], basic fibroblast growth factor [bFGF], and epidermal growth factor [EGF]) and muscarinic agonists induce neurite outgrowth via distinct intracellular pathways that cross-talk with each other. The agonist, via activation of M1 mAChR, restores learning and memory impairments with an excellent safety margin, mediate Aβ processing, and decrease τhyperphosphorylation, and thus may be useful in Alzheimer’s disease therapy [26]. In a recent animal study, a novel compound was reported, AF710B, a highly potent and selective allosteric M1 muscarinic and σ1 receptor agonist. AF710B exhibits an allosteric agonistic profile on the M1 muscarinic receptor; very low concentrations of AF710B significantly potentiated the binding and efficacy of carbachol on M1 receptors and their downstream effects (p-ERK1/2, p-CREB). The σ1 receptor (σ1R) is another potential target for drug development for AD, as it is considered to play a fundamental role in cognitive function. The compound could (i) mitigate cognitive impairments in the Morris water maze and (ii) decrease BACE1, SK3β activity, p25/CDK5, neuroinflammation, soluble and insoluble Aβ, Aβ, plaques and tau pathologies [29].

SS patients also were found to have significantly lower basal cortisol levels compared to healthy controls, which is thought to result from hypothalamic–pituitary–adrenal axis (HPA) deterioration [30, 31]. Le et al. [31] showed a significant correlation between cortical hypoperfusion and cognitive dysfunction. Another mechanism possibly linking SS to AD is the autoantibodies produced in SS, which may play a role in AD development. These autoantibodies can form an immune complex, and subsequently injure the endothelial walls of small blood vessels [19]. These studies further suggest that inflammation likely contributes to the pathogenesis of accelerating neurodegeneration among SS patients [19, 32].

We also found that, in comparison with patients without SS, primary SS patients were more likely to have hyperlipidemia, hypertension, and CAD. These factors also may explain the higher portion of AD in this cohort [9–11]. Our findings match the prevalence community-based reports that indicate that primary SS patients have higher cardiovascular risk factors at primary SS diagnosis. Akyel et al. [11] reported that myocardial function is disturbed in SS patients and there is significant atrial electromechanical delay. Our findings support their observation, in that we found a higher prevalence of CAD. Therefore, cardiovascular risk factors should be taken into account when managing such patients.

Autoimmune-induced dementia is an acute or sub-acute disorder that affects memory, cognition, and behavior. More importantly, autoimmune dementia is reversible and may be misdiagnosed as an irreversible neurodegenerative disorder [12, 13]. However, the true incidence rate of autoimmune dementia is unknown; therefore, it is difficult to assign correct ICD-9-CM codes to autoimmune dementia. A review by Rossor et al. [33] showed that young onset dementia had a higher prevalence of genetic or metabolic diseases. Most autoimmune dementia occurs in patients younger than 45 years, and AD is far more common in the geriatric population [34]. Therefore, different age groups were used to check and prevent misclassification errors. In the present study, most patients with primary SS and AD were more than 70 years old. The diagnosis of AD is highly suitable.

One major strength of our study was that it was based on a nationwide, population-based, case cohort study, which minimized the problems of insufficient power. Furthermore, the potential for selection biases was minimized by the comprehensive coverage of the NHI system and a large sample size.

Nevertheless, there are several limitations in our study. First, the diagnosis of primary SS and AD was made using the ICD-9 code from the database. NHIRD does not provide personal information, such as smoking preference, education level, and body mass index. Intelligence and education level also may have some effect on the occurrence of AD. These co-variables could not be adjusted in the analysis. Second, NHIRD provides limited information on clinical characteristics, such as disease severity, laboratory data, and imaging information. Therefore, it is hard to know the relationship between clinical severity and brain region involvement in primary SS and AD. Finally, the incidence rate of AD may be underestimated because inpatient claims data were used.

In conclusion, our nationwide cohort study found that patients with primary SS have a higher prevalence of AD than patients without primary SS. Although the evidence suggests that primary SS is a risk factor for AD, very little is known about the duration or clinical severity of primary SS or how it may contribute to AD. Therefore, further research in this area is necessary to answer such questions, and to examine whether proper management of primary SS may help prevent the occurrence of AD.
